# Expression Quantitative Trait Loci Information Improves Predictive Modeling of Disease Relevance of Non-Coding Genetic Variation

**DOI:** 10.1371/journal.pone.0140758

**Published:** 2015-10-16

**Authors:** Damien C. Croteau-Chonka, Angela J. Rogers, Towfique Raj, Michael J. McGeachie, Weiliang Qiu, John P. Ziniti, Benjamin J. Stubbs, Liming Liang, Fernando D. Martinez, Robert C. Strunk, Robert F. Lemanske, Andrew H. Liu, Barbara E. Stranger, Vincent J. Carey, Benjamin A. Raby

**Affiliations:** 1 Channing Division of Network Medicine, Department of Medicine, Brigham and Women’s Hospital and Harvard Medical School, Boston, Massachusetts, United States of America; 2 Division of Pulmonary and Critical Care Medicine, School of Medicine, Stanford University, Stanford, California, United States of America; 3 Program in Translational NeuroPsychiatric Genomics, Department of Neurology, Brigham and Women’s Hospital, Boston, Massachusetts, United States of America; 4 Division of Genetics, Department of Medicine, Brigham and Women’s Hospital and Harvard Medical School, Boston, Massachusetts, United States of America; 5 Program in Medical and Population Genetics, Broad Institute, Cambridge, Massachusetts, United States of America; 6 Departments of Biostatistics and Epidemiology, Harvard School of Public Health, Boston, Massachusetts, United States of America; 7 Arizona Respiratory Center and BIO5 Institute, University of Arizona, Tucson, Arizona, United States of America; 8 Division of Allergy, Immunology and Pulmonary Medicine, Department of Pediatrics, Washington University School of Medicine, St. Louis, Missouri, United States of America; 9 University of Wisconsin School of Medicine and Public Health, Madison, WI, United States of America; 10 Division of Allergy and Clinical Immunology, Department of Pediatrics, National Jewish Health and University of Colorado School of Medicine, Denver, Colorado, United States of America; 11 Section of Genetic Medicine, Department of Medicine, University of Chicago, Chicago, Illinois, United States of America; 12 BWH Pulmonary Genetics Center, Division of Pulmonary and Critical Care Medicine, Department of Medicine, Brigham and Women's Hospital and Harvard Medical School, Boston, Massachusetts, United States of America; Cincinnati Children's Hospital Medical center, UNITED STATES

## Abstract

Disease-associated loci identified through genome-wide association studies (GWAS) frequently localize to non-coding sequence. We and others have demonstrated strong enrichment of such single nucleotide polymorphisms (SNPs) for expression quantitative trait loci (eQTLs), supporting an important role for regulatory genetic variation in complex disease pathogenesis. Herein we describe our initial efforts to develop a predictive model of disease-associated variants leveraging eQTL information. We first catalogued *cis*-acting eQTLs (SNPs within 100kb of target gene transcripts) by meta-analyzing four studies of three blood-derived tissues (*n* = 586). At a false discovery rate < 5%, we mapped eQTLs for 6,535 genes; these were enriched for disease-associated genes (*P* < 10^−04^), particularly those related to immune diseases and metabolic traits. Based on eQTL information and other variant annotations (distance from target gene transcript, minor allele frequency, and chromatin state), we created multivariate logistic regression models to predict SNP membership in reported GWAS. The complete model revealed independent contributions of specific annotations as strong predictors, including evidence for an eQTL (odds ratio (OR) = 1.2–2.0, *P* < 10^−11^) and the chromatin states of active promoters, different classes of strong or weak enhancers, or transcriptionally active regions (OR = 1.5–2.3, *P* < 10^−11^). This complete prediction model including eQTL association information ultimately allowed for better discrimination of SNPs with higher probabilities of GWAS membership (6.3–10.0%, compared to 3.5% for a random SNP) than the other two models excluding eQTL information. This eQTL-based prediction model of disease relevance can help systematically prioritize non-coding GWAS SNPs for further functional characterization.

## Introduction

The vast majority (88%) of complex disease-associated single nucleotide polymorphisms (SNPs) identified by genome-wide association studies (GWAS) are non-coding variants [1]. Genomic analyses of these SNPs, or their proxies in strong linkage disequilibrium (LD), find significant enrichment for putative functional regulatory regions that can affect the expression of nearby genes [2–4], further supporting an important role for regulatory genetic variation in disease pathogenesis and motivating extensive cataloging of such variation [5]. In contrast to disease-associated variants localized to the coding regions of gene transcripts, distinguishing functionally relevant non-coding variants from their more numerous irrelevant counterparts is considerably more challenging [6].

In particular, expression quantitative trait locus (eQTL) mapping, a genetic method that relates SNP allelic variation to target transcript abundance [7], could provide valuable information for prioritizing disease GWAS results. Performed in diverse tissues and cell types, eQTL studies have identified thousands of regulatory variants that, on average, individually explain ~10% of population variability in gene expression at each locus [8], and are collectively significantly enriched for disease-associated variants [2, 3, 8–10]. Given that there are multiple lines of genomic evidence for the functionality of eQTLs [11], we propose that improved prioritization of non-coding genetic variation reported in disease-association mapping studies can be achieved by combining SNP-specific eQTL information together with other relevant annotations, such as putative regulatory chromatin states [12], to develop multivariate prediction models. Herein we describe such an approach.

An important first step in the development of a high-performing model is ensuring the accuracy of the variables (i.e., sequence features) being considered as model predictors. In the case of eQTL data, a major concern relates to the statistical power to detect such associations. Though the effects of SNPs on gene expression variability are typically much stronger than their downstream effects on trait liability [7], like all genetic studies, eQTL analyses are often limited in their statistical power; heritability estimates in twin studies suggest that a substantial proportion of the total genetic variability of gene expression remains unexplained [8, 9]. Indeed, the yield of individual eQTL studies is strongly correlated with study sample size, with the greatest number of variants identified in the few studies that include thousands of subjects [9, 13]. Given the increasing availability of results from eQTL studies, meta-analysis of smaller existing datasets is a natural solution for increasing power to identify additional regulatory variants. Descriptions of the many technical considerations of eQTL meta-analytic approaches have been reported [14–18], including current hurdles for novel eQTL discovery using already published datasets [19]. In this study, we meta-analyzed data on 586 subjects from four cohorts to identify *cis*-eQTLs among three blood-derived tissues.

Information on the strength of association for these blood-derived eQTLs was then used in conjunction with other genomic SNP annotations to develop predictive models of disease relevance. While all the resulting models improved our ability to distinguish disease-associated variants from others, the model that included eQTL association information out performed those that did not, demonstrating the utility of eQTL information in prioritizing for further study non-coding genetic variation associated with complex diseases.

## Materials and Methods

### Expression and genotype data

Expression and genotype data from four cohorts of unrelated individuals of non-Hispanic European ancestry were studied: peripheral blood CD4+ lymphocytes (CD4) sampled from 80 individuals with asthma participating in the Childhood Asthma Research and Education Network (CARE) [20]; lymphoblastoid cell lines (LCL) from 115 individuals in the Centre d'Etude du Polymorphisme Humain (CEPH) International HapMap Project population (CEU) [21]; and CD4+ cells (*n* = 200) and whole blood (WB) samples (*n* = 216) from two subsets of asthmatics participating in the Childhood Asthma Management Program (CAMP) [22]. The CARE CD4 and CAMP WB expression data were generated using Illumina HT12 arrays (v3 and v4, respectively; Illumina, Inc., San Diego, CA), as part of the Asthma BioRepository for Integrative Genomic Exploration (W. Qiu *et al*., 2012, American Thoracic Society, abstract). The CAMP CD4 and CEU LCL expression data were generated using Illumina Human-Ref8 v3 BeadChip arrays [3, 23]. We identified 12,889 expression probes that were (1) represented on both platforms and testable in all four populations, (2) located on autosomes, (3) mapped uniquely to the genome, (4) not affected by any known SNP-under-probe effect, and (5) considered either “Good” or “Perfect” using the Illumina annotation algorithm described by Barbosa-Morais *et al*. [24]. Approval was obtained from the Institutional Review Boards (IRB) of Brigham and Women's Hospital (Boston, MA) and each of the participating institutions for CAMP and CARE. Written informed consent was obtained from those study participants.

Detailed methods of genome-wide SNP genotyping have been described elsewhere for CEU [25], CARE [26], and CAMP [3, 27]. A common set of SNP genotypes was obtained by imputation in each cohort using MaCH (version 1.0) [28] and the 1000 Genomes Project EUR reference phased haplotypes based on Phase 1 low coverage data (20101123 release). For fifty-two CEU individuals directly sequenced as part of the 1000 Genomes Project, we substituted actual genotype data in place of imputed data. SNPs with minor allele frequency (MAF) < 1%, a Hardy-Weinberg equilibrium *P* < 0.001, and/or an imputation quality score < 0.3, were excluded, resulting in a set of ~37 million variants per cohort. We performed principal component analysis (PCA) of the genotypes in each cohort using EIGENSOFT (version 3.0) [29, 30]. Genetic outliers identified based on Tracy-Widom statistics computed on the genotype PCs by the accompanying utility TWSTATS [30] were removed from further analysis. The total numbers of remaining individuals were thus *n* = 73 for CARE CD4, *n* = 113 for CEU LCL, *n* = 198 for CAMP CD4, and *n* = 202 for CAMP WB.

### Association testing

The gene expression data were first quantile-normalized across the four cohorts and adjusted for age, gender, and known batch variables. To account for unmeasured confounders, within each population, we empirically determined the number of gene expression principal components (PCs) to adjust for in order to maximize the number of nominally significant eQTL associations identified (*P* < 0.05). SNPs were iteratively tested for associations with age- and gender-adjusted expression residuals with increasing numbers of gene expression PCs. The expression PCs corresponding to the 21, 19, 31, and 32 largest eigenvalues in CARE CD4, CEU LCL, CAMP CD4, CAMP WB, respectively, were thus adjusted for using the “clipPCs” function from the R package “GGBase” (version 3.24) from Bioconductor (release 2.14) [31], which provides base programmatic infrastructure for the study of the genetics of gene expression. The expression and genotype datasets for each cohort were then bundled together into “smlSet” data objects to conveniently facilitate downstream analyses in a unified Bioconductor workflow within R (version 3.1). Access to the source genotype and expression datasets for the CAMP and CARE cohorts must be approved by the corresponding study IRBs.

To systematically assess the genotype-expression association landscape in *cis* for each gene (probe), all SNPs within a search radius of 100 kb from a given pair of target transcript flanks were tested for evidence of being an eQTL. This search was facilitated by the "All.cis" function from the R package “GGtools” (version 4.10) [32] from Bioconductor, which provides analytical tools for the study of the genetics of gene expression. The gene boundaries were based on the “knownGene” track downloaded from the University of California Santa Cruz (UCSC) Genome Browser [33]. Each cohort-specific SNP-probe association was represented by a one degree-of-freedom *χ* ^2^ test statistic.

We then performed a meta-analysis of the *cis*-eQTL associations from all four populations by summing together their corresponding test statistics into a single four degrees-of-freedom *χ* ^2^ test statistic. In all association analyses, a false discovery rate (FDR) for each eQTL association was estimated by permutation testing (*k* = 3 permutations) using an implementation of a plug-in FDR methodology (Algorithm 18.3) [34]. Briefly, the plug-in FDR algorithm obtains hundreds of millions of realizations of the null distribution of the association statistic through multiple genome-wide permutations of genotype against expression for all SNP-gene pairs in *cis*. Tail probabilities for extreme values of the observed statistic are estimated accurately and realistically from this ensemble of realizations of the null distribution and provide our estimates of the FDRs. Gene-wise FDRs optimized over all SNPs *cis* to each gene were thus calculated using the "collectFiltered" function from GGtools. The overlaps among the meta-analysis cohorts of the sets of genes with significant eQTLs were visualized using functions from the R package “VennDiagram” (version 1.6). To evaluate the replicability of the meta-analysis results, a list of previously published eQTL genes was downloaded from the supplementary materials of a study in WB by Westra *et al*. [13].

### GWAS enrichment and prediction analyses

To assess the relationship of the observed meta-analysis eQTL genes with known GWAS genes, we calculated the proportion of GWAS genes harboring a least one significant meta-analysis eQTL SNP (FDR < 5%). As input, we obtained a set of 12,161 gene identifiers reported in 9,764 entries in the National Human Genome Research Institute (NHGRI) GWAS Catalog (accessed 28 November 2012, https://www.genome.gov/gwastudies/) [1], Identifiers listed in the NGHRI GWAS Catalog as “genes” but not matching any gene names found in the UCSC Genome Browser were removed, including instances of “intergenic” (*n* = 1,091), “NR” for “Not Reported” (*n* = 685), “pseudogene” or other terms that indicated a non-genic locus, and non-standard gene identifiers, resulting in a final set of 4,250 unique GWAS genes. Enrichment and its significance was determined by comparing the observed proportion of eQTL genes among a given test gene set to a null distribution of 10,000 draws of the same number of random genes from across the genome (represented by a set of 19,058 UCSC Genome Browser genes). We performed enrichment analyses of manually curated candidate subsets of genes associated with inflammatory diseases and traits, metabolic traits, mental health traits, cancers, and adult height.

To predict whether a given SNP (and its target probe) was in the set of GWAS index SNPs or their close proxies from the NHGRI GWAS Catalog, we developed a set of three multivariate generalized logistic regression models in R incorporating different combinations of SNP annotation information in the forms of factored variables related to the genomic distance of a given SNP from the transcript boundaries of its target gene, its MAF bin, its eQTL FDR bin, and its predicted regulatory chromatin state in GM12878 LCLs based on genomic data from the Encyclopedia of DNA Elements (ENCODE) [12]. To develop a training set of eQTLs for the model, a set of 381,491 independent SNPs from across the genome was identified in the EUR panel of the 1000 Genomes Project (Phase 1, version 3) using the default settings for variance inflation factor-based pruning implemented in PLINK (version 1.07) [35]. These SNPs were located in *cis* to 11,864 unique probes, resulting in 777,998 SNP-probe pairs in the training set. To compare their relative contributions, the effect betas from the training set of each predictive model were plotted using the “forestplot” function from the R package “rmeta” (version 2.16), which provides tools for visualizing meta-analysis results. Corresponding receiver operating characteristic (ROC) curves for the predictive models were generated using the “roc” function from the R package “pROC” (version 1.8) [36], which provides tools for visualizing and analyzing ROC curves. For an additional comparison, scores from version 1.0 of the Combined Annotation Dependent Depletion (CADD) [37] resource were also included as a model predictor. The scaled CADD scores were turned into factored variables based on the following bins: [0, 5], (5,10], (10, 20], (20,30], (30, 60].

## Results

The goal of this study was to develop a multivariate prediction model to prioritize the further study of non-coding genetic variation with greatest relevance to disease pathogenesis. We based this model on several relevant SNP annotation predictors, including distance from target gene transcript, minor allele frequency, chromatin states, and association evidence for being a *cis*-eQTL. To optimize the quality of our eQTL information, we first conducted a meta-analysis of eQTL associations from four cohorts representing three blood-derived tissues (*n* = 586 subjects). We first describe the results of this meta-analysis, followed by the results of our prediction model building.

### eQTL meta-analysis yields and performance

Compared to the eQTL analysis of each cohort individually, the meta-analysis considering all 586 individuals resulted in substantial gains in the number of regulatory variants identified (Table 1). In contrast to the individual cohort analyses, where we identified between 626 and 5,363 unique genes with significantly associated eQTLs (corresponding to between 21,415 and 366,077 significantly associated pairs of SNPs and their respective target gene expression probes at an FDR < 5%, *P* = 2.6 × 10^−07^ to 3.6 × 10^−04^), the meta-analysis identified a total of 6,535 eQTL genes (488,290 SNP-probe pairs, *P* = 1.9 × 10^−81^ to 3.5 × 10^−03^). A quantile-quantile plot of the meta-analysis associations showed a very substantial enrichment in the right-hand tail of the observed distribution above the permuted null distribution (S1 Fig). The full meta-analysis and cohort-specific eQTL results have been made available on-line (https://regepi.bwh.harvard.edu/projects/eQTLMeta/).

**Table 1:** Summary characteristics of cohort-specific and meta-analysis eQTL results.

	CARE CD4	CEU LCL	CAMP CD4	CAMP WB	META
**Sample Size**	73	113	198	202	586
**Total # SNPs**	4,253,296	4,119,125	4,243,510	4,260,335	3,804,162
**# Signif. SNPs**	20,577	60,217	319,846	229,849	421,377
**Total # Genes**	11,121	10,924	11,121	11,121	10,924
**# Signif. Genes**	626	2,184	5,363	4,155	6,535
**Total # SNP-Probe Pairs**	9,153,510	8,305,457	9,138,022	9,168,425	7,672,940
**# Signif. SNP-Probe Pairs**	21,415	64,491	366,077	254,186	488,290
**Min. Signif. Nominal P Value**	1.84E-12	1.22E-21	2.62E-31	2.58E-37	1.95E-81
**Max. Signif. Nominal P Value**	4.43E-04	1.44E-03	3.57E-04	2.26E-03	3.53E-03

Total numbers of SNPs only include those located within 100 kb of target gene trancripts. Multiple probes may target the same gene. In each of the four study cohorts (“CARE CD4”, “CEU LCL”, “CAMP WB”, and “CAMP CD4”) and in their combined meta-analysis (“META”), eQTL association significance was defined as an FDR < 5% (see *Materials and Methods*). The corresponding minimum and maximum uncorrected *P* values are also reported.

**Fig 1:**
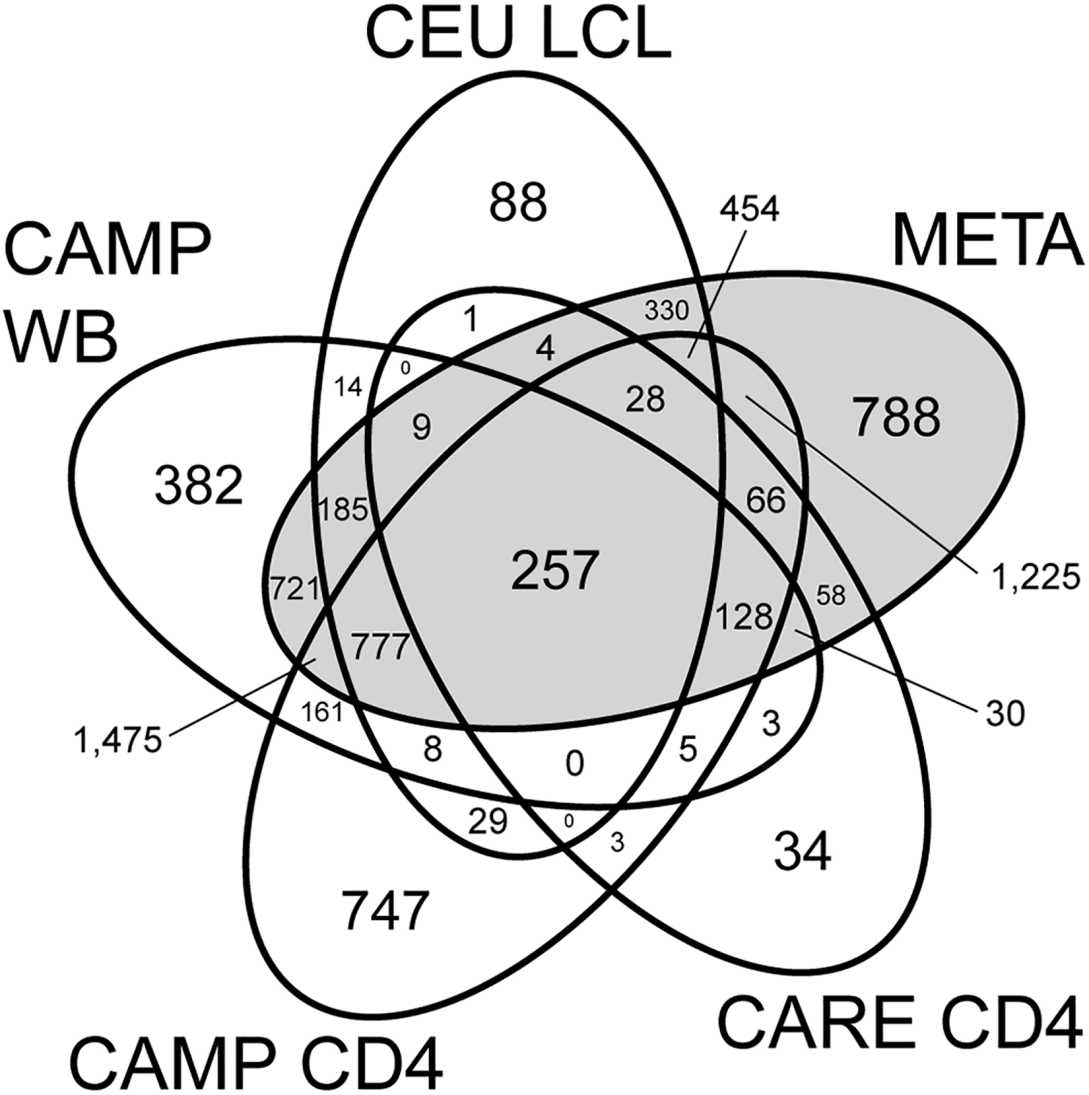
Venn diagram of overlaps of eQTL genes identified in specific individual cohorts and through meta-analysis. Numbers represent counts of genes with at least one significant eQTL SNP (FDR < 5%) in each of the four study cohorts (“CAMP WB”, “CAMP CD4”, “CEU LCL”, and “CARE CD4” in white ellipses) and in their combined meta-analysis (“META” in gray ellipse). Total counts for each group are also summarized in **Table 1**.

**Fig 2:**
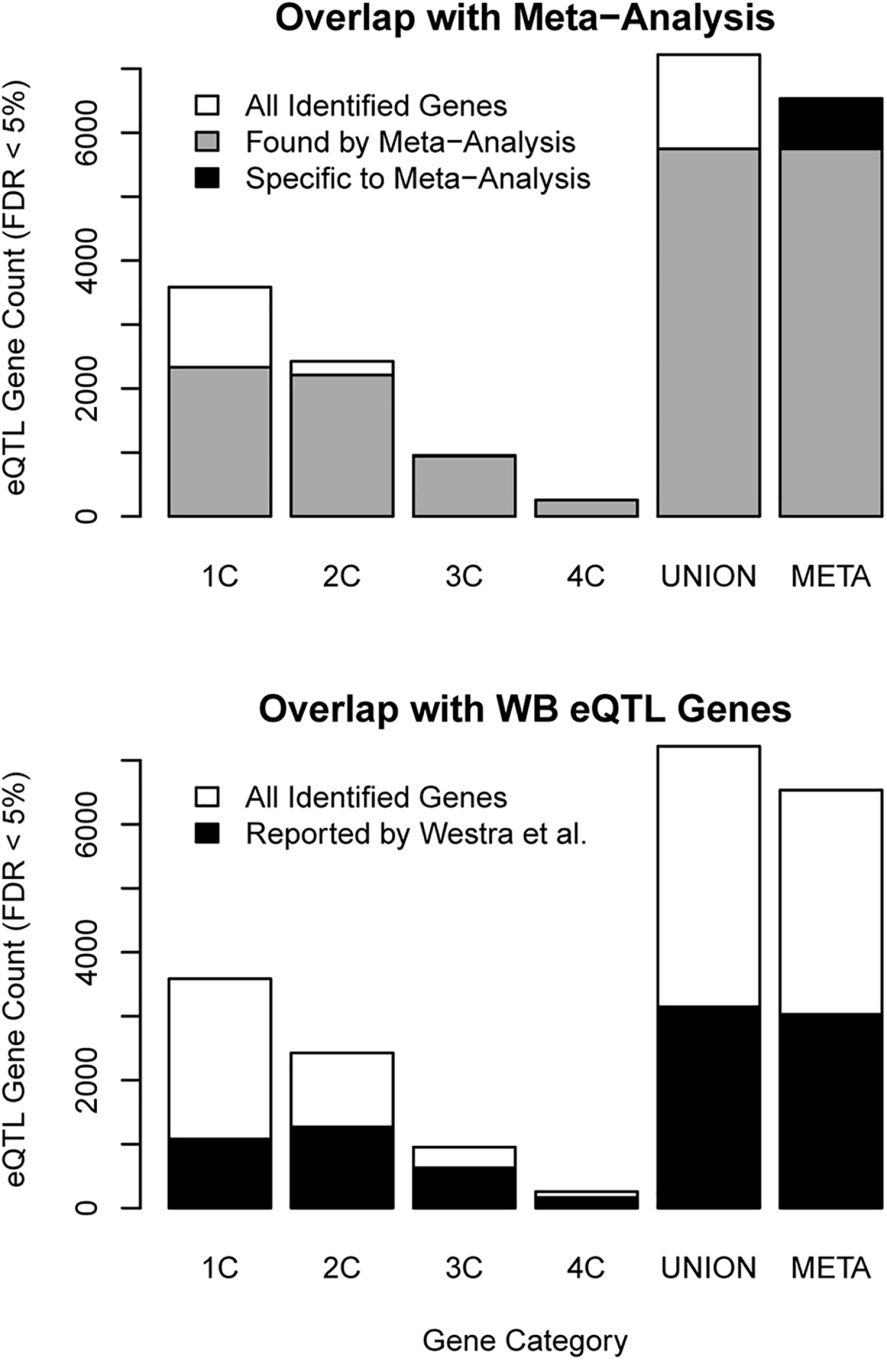
Relationships of eQTL meta-analysis gene yields with representation in individual cohorts and a previous study. Counts of all significant eQTL genes (meta-analysis FDR < 5%, **Table 1**) identified per source category are shown with white bars. The first four categories (“1C” through “4C”) represent the number of individual cohorts in which a gene was identified. The fifth category (“UNION”) is the union of the genes from the preceding four categories. The sixth category (“META”) is the set of genes identified in the meta-analysis. *Top panel*: For comparison, the counts of genes in each category also found by the meta-analysis are shown with overlapping gray bars. Among genes found in the meta-analysis, the count of genes not identified in any of the individual cohorts is shown with a black bar. *Bottom panel*: The counts of genes found in an eQTL study in WB by Westra *et al*. [13] are shown with black bars.

The individual cohorts together implicated 7,222 unique eQTL genes and an additional 788 were identified only through the subsequent meta-analysis (Figs 1 and 2). Among these total 8,010 eQTL genes identified, the strongest evidence existed for the 257 genes that were detected in each of the four cohorts and also by the by meta-analysis (Fig 1). Of the remainder, 3,156 genes were detected in two or three cohorts and in the meta-analysis; 2,334 were unique to one cohort and also detected in the meta-analysis (Fig 2). A total of 1,475 genes were only detected in individual cohorts but were not significant in the meta-analysis. Of these, 1,251 (85%) were observed in only one of the four cohorts, suggesting they may represent either false positive results or tissue-specific eQTL signals drowned out by the meta-analysis.

We then evaluated the replicability of the 6,535 eQTL genes detected in our meta-analysis by examining their overlap with 6,368 genes reported in a substantially larger eQTL study (*n* = 5,311 samples) of whole blood by Westra *et al*. [13]. Overall, 3,040 (46%) of 6,535 genes detected in our meta-analysis were also reported by Westra *et al*. (Fig 2). The degree of overlap between the two studies was greatest for those eQTL genes with greatest evidence of association in the meta-analysis and conversely declined with the strength of association. Genes with an FDR < 5% in the meta-analysis and in all four individual cohorts showed 65% overlap with Westra *et al*. Genes detected by meta-analysis and in only one cohort showed 49% overlap. Finally, overlap was lowest (20%) for those genes detected in only one cohort but not detected by meta-analysis. Notably, overlap was greater for eQTL genes detected by meta-analysis alone (26%) compared to those detected only in one cohort (20%), suggesting that even the weakest category of meta-analysis results were more often replicated than the results from only single cohorts.

### The relationship of eQTLs to disease-associated loci

**Fig 3:**
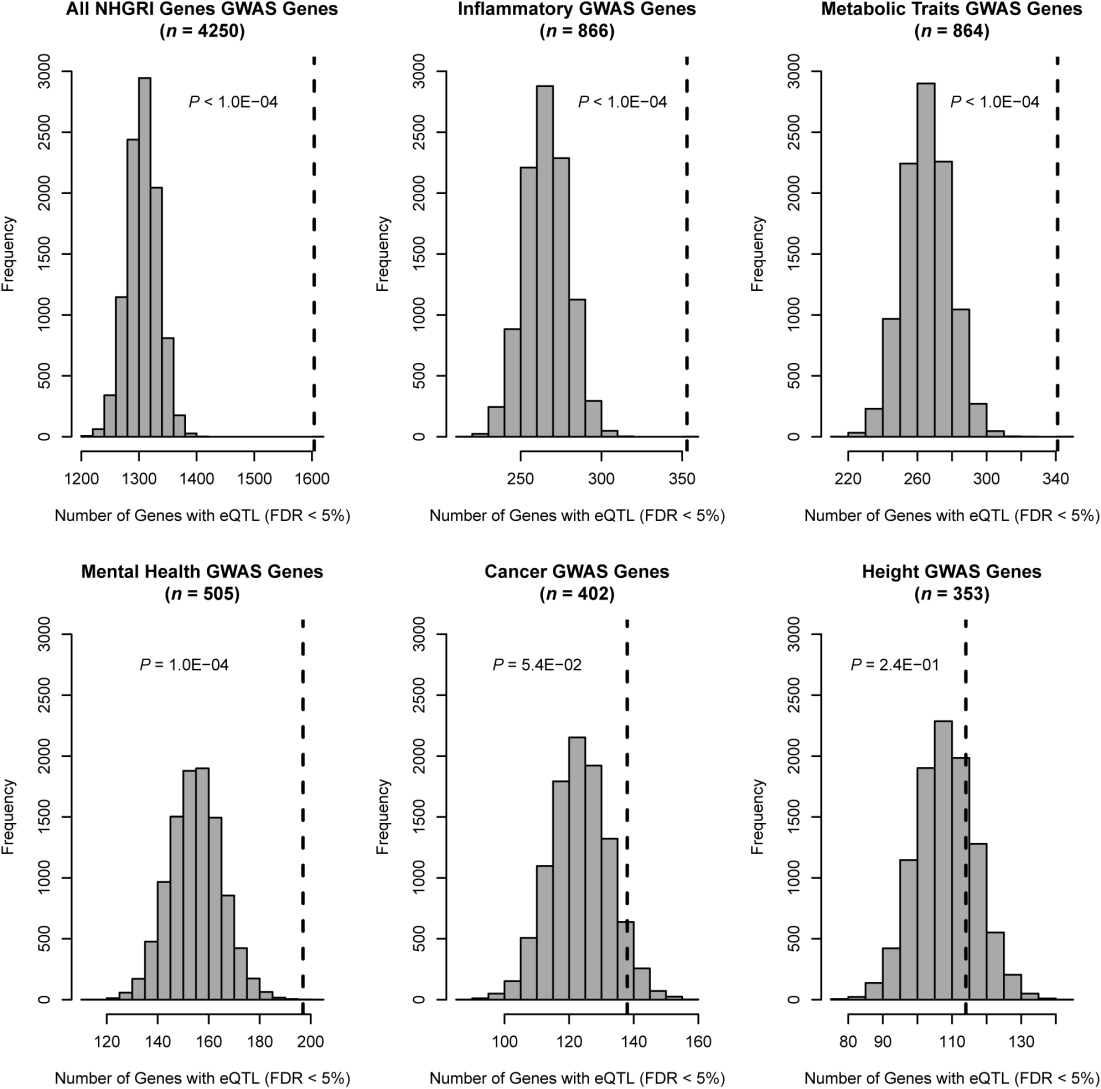
Genes associated with inflammatory and other categories of disease traits enriched for meta-analysis eQTL genes. In each histogram, the observed number of genes in the given category harboring at least one significant eQTL SNP (meta-analysis FDR < 5%) is marked with a dashed vertical line. The null distributions derived from 10,000 permutations are shown with gray bars.

Given the reported enrichments of disease-associated variants for eQTLs [2, 3, 9] and for other functional sequence annotations [11, 12], we next assessed the relationship of the eQTL genes detected in the meta-analysis with disease-associated genes by comparing their representation in the NHGRI GWAS Catalog. Among the 6,535 eQTL genes detected by meta-analysis, 950 (14.5%) were previously reported as GWAS loci, representing a significant enrichment among GWAS genes those that harbor at least one eQTL (*P* < 10^−04^) (Fig 3). This enrichment appeared to be most robust for genes associated with inflammatory, metabolic and mental health traits. Though we have previously reported associations with height- and cancer-associated regulatory variants detected in CD4+ lymphocytes [3], there were no significant enrichment for meta-analysis eQTLs for these disease categories (*P* ≥ 0.05), possibly reflecting the larger proportion of samples from peripheral blood contributing to the meta-analysis.

### eQTL information for improved disease SNP prediction

**Fig 4:**
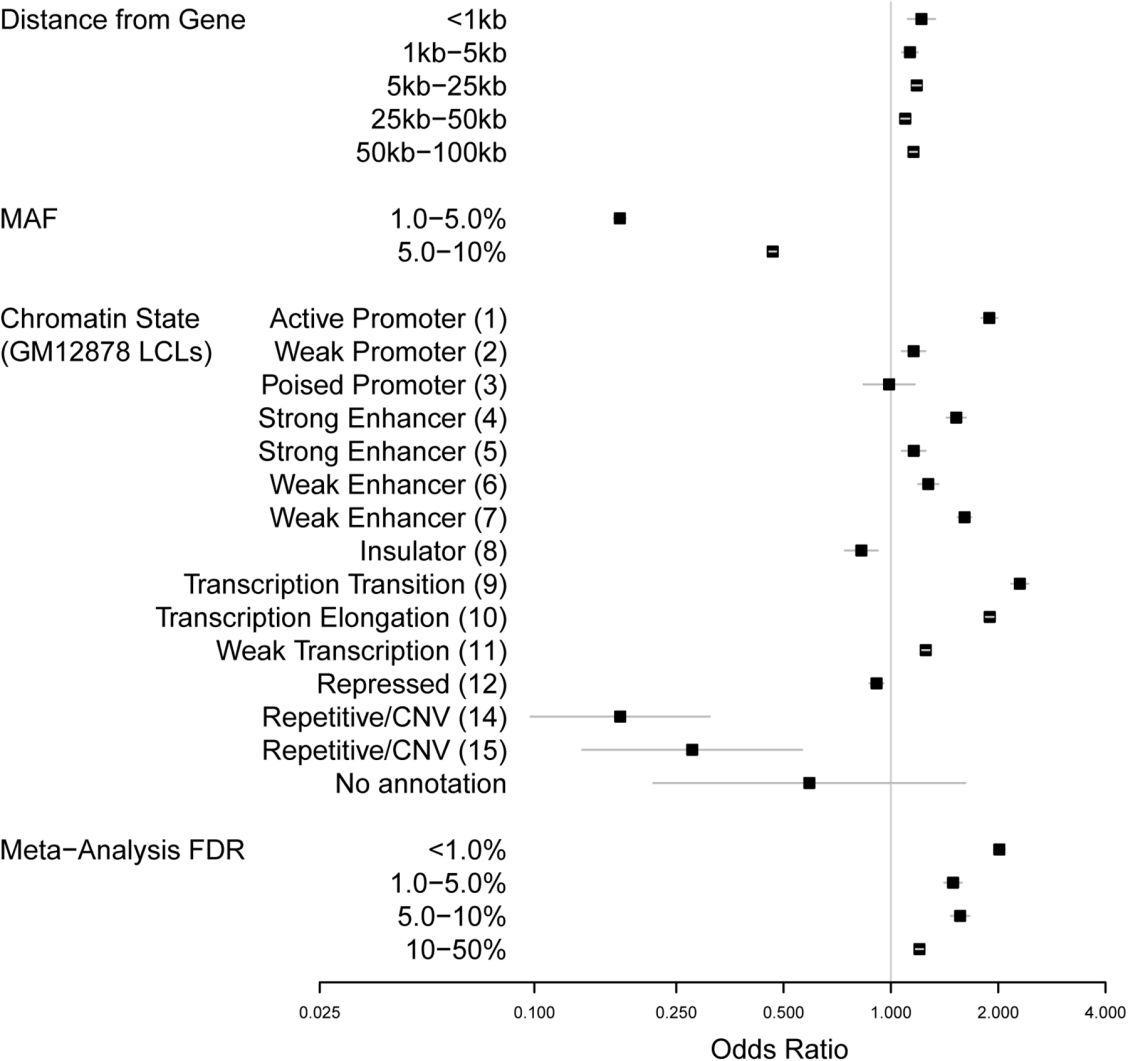
Forest plot of component effects of complete GWAS predictive model based on training set of SNPs. Odds ratios (black squares) from the complete multivariate model (“chromstate+eqtl [M3]”) for features predicting the membership of a SNP in the NHGRI GWAS Catalog are shown here with standard errors (gray lines). Smaller models are shown for comparison in S2 Fig. Four classes of SNP annotation are represented in the model, each with multiple levels: distance from gene, MAF, chromatin state in GM12878 LCLs (12), and evidence of eQTL association based on meta-analysis FDR. The base levels for each annotation are “0 kb (within gene)” [Distance from Gene], “>10%” [MAF], “Heterochromatin (13)” [ChromHMM], and “>50%” [FDR].

We next developed multivariate logistic predictive models of the likelihood of a SNP being a “GWAS hit”, namely being reported in the GWAS Catalog (or having a close proxy at *r* ^2^ > 0.8). The complete model (“chromstate+eqtl [M3]”) considered multiple SNP features, including physical distance from target transcript, MAF, putative chromatin state in LCLs [12], and strength of eQTL association as estimated from our meta-analysis (Fig 4). For comparison, we also examined two smaller models: one that considered physical distance from target transcript, MAF, and the variants’ position relative to transcript (“structure [M1]”) and one that considered physical distance from target transcript, MAF, and the chromatin state annotations (“chromstate [M2]”) (S2 Fig). MAF was included in all models to adjust for the overrepresentation of common variants in the GWAS Catalog, which is a reflection of the inherent power-related bias of GWAS to detect associations with common variants.

**Fig 5:**
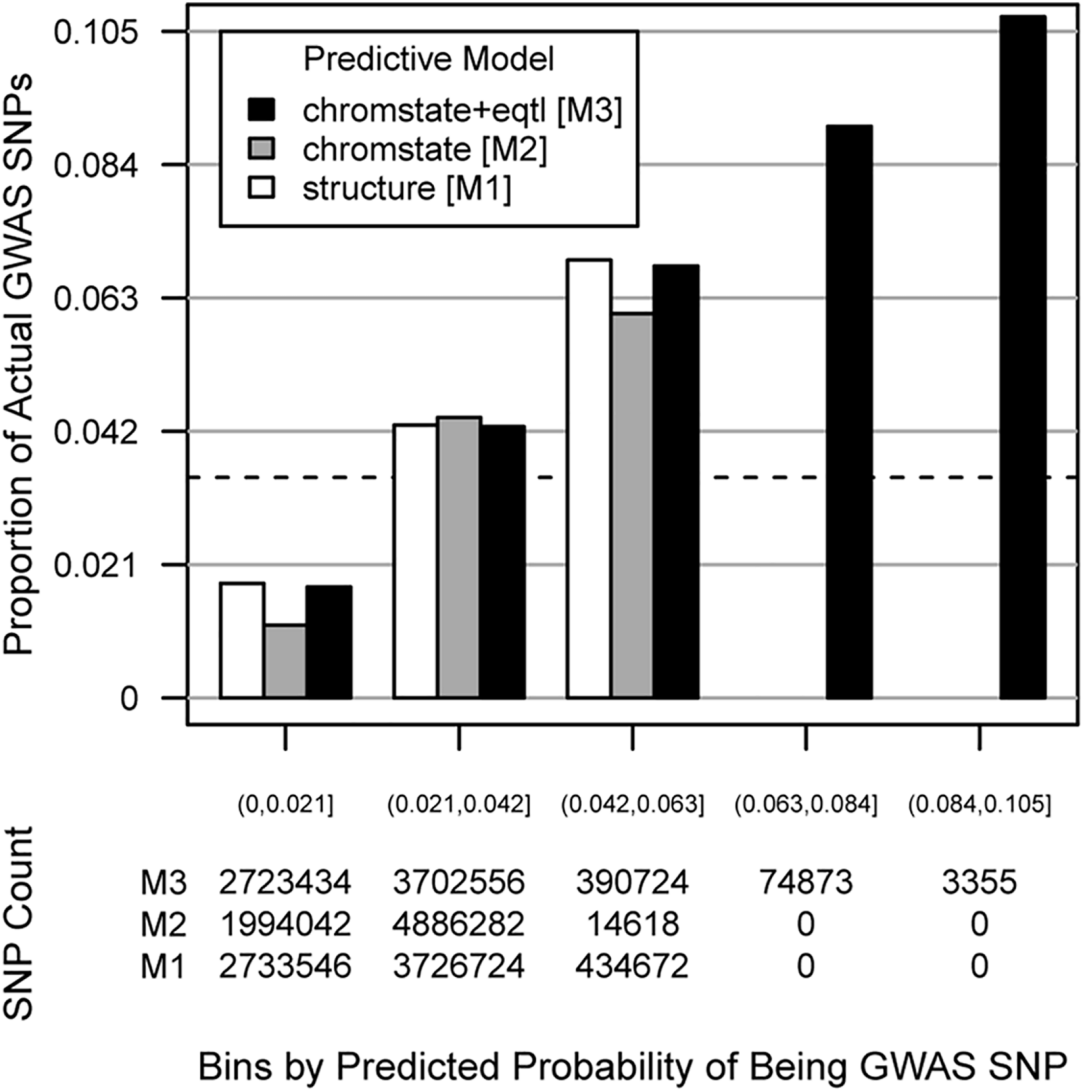
Multivariate logistic models predicting SNP membership in GWAS are well-calibrated. Top panel: Three models were developed for predicting the membership of a given SNP in the NHGRI GWAS Catalog, all incorporating at minimum the distance of the SNP from the transcript boundaries of its target gene and the minor allele frequency of the SNP. The "structure [M1]" model (white) also incorporates the NCBI gene structure classification of the gene (intron, coding, untranslated region, etc.) (S2 Fig); "chromstate [M2]" (gray) instead incorporates chromatin state (S2 Fig); "chromstate+eqtl [M3]" (black) incorporates both chromatin state and eQTL FDR class (Fig 4). The *x*-axis shows equal-sized bins of predicted probabilities of being a GWAS SNP. This particular choice of bins based on the widest range of probabilities (from M3) aids visual comparison of calibration among the three models by smoothing the proportions of observed GWAS SNPs. The *y*-axis shows the actual proportion of GWAS SNPs in that bin. The dashed green line at 3.5% represents the mean probability of a random SNP in the genome for being a GWAS hit or a close proxy (*r* ^2^ > 0.8) for one. Bottom panel: a table of absolute counts of SNPs in each predicted probability bin for each of the predictive models. For the M1 and M2 models, no SNPs had predicted probabilities > 6.3%.

**Fig 6:**
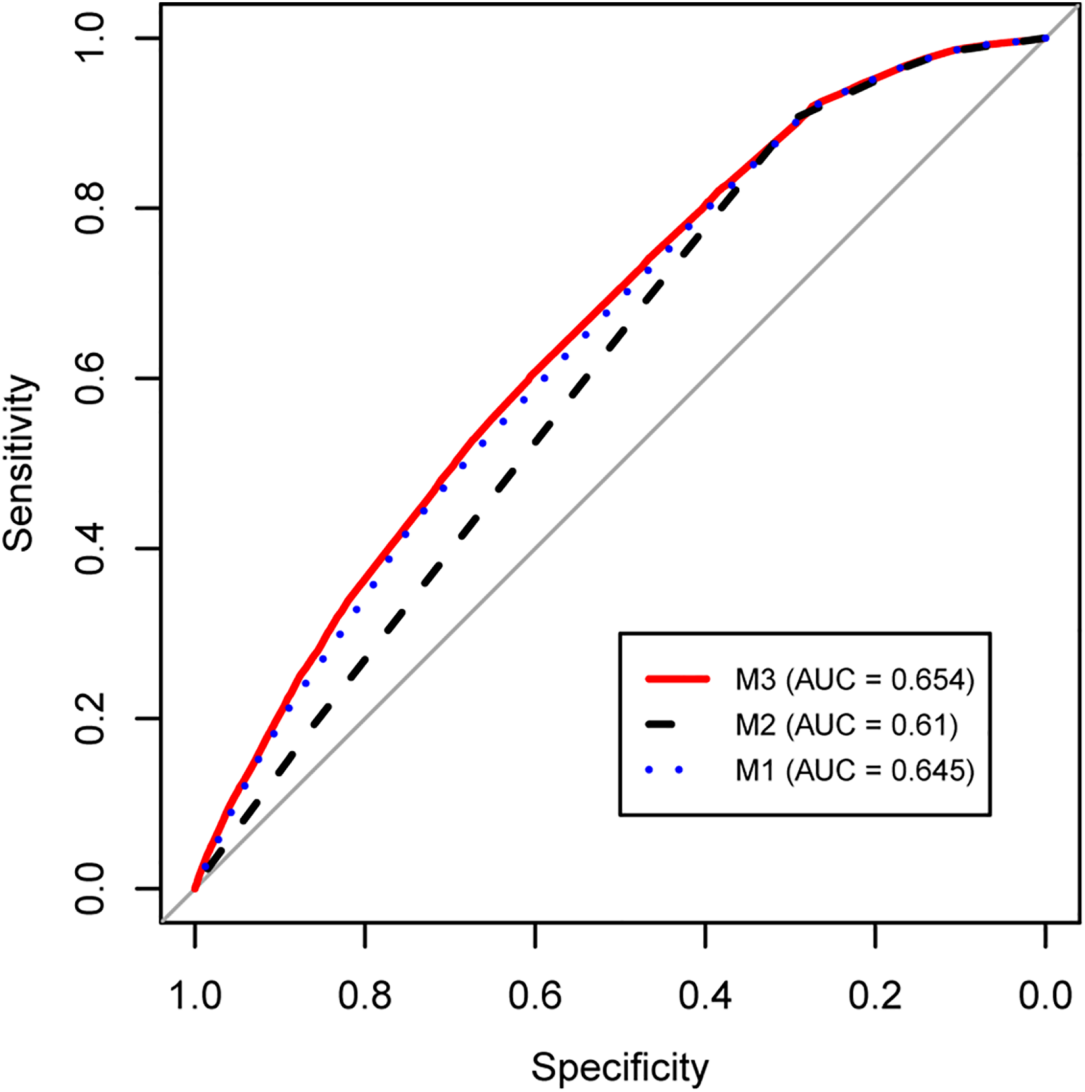
ROC curves for multivariate logistic models predicting SNP membership in GWAS. Components of the three predictive models are described in Fig 5.

We trained these three predictive models on the same random subset of 777,998 SNP-probe pairs, and then validated the predictive power of each model by testing against the remaining 6,894,942 SNP-probe pairs that were not included in the training set. While a randomly selected SNP-probe pair in the test set had a 3.5% chance of being a GWAS hit, all three models predicted substantial subsets of pairs to have even higher probabilities (Fig 5). All three models were well-calibrated in that for SNP-probe pairs found in a given bin of predicted probabilities, the actual observed proportion of GWAS hits among those pairs was within that predicted range. However, our complete model M3 that considered both eQTL evidence and chromatin state outperformed the smaller models M1 and M2 in one important regard: whereas the maximum predicted probabilities generated by M1 and M2 peaked at 6.0% and 5.3%, respectively (corresponding to maximum 1.7-fold and 1.5-fold chances, respectively, of being a GWAS hit compared to random), M3 derived probabilities had a greater dynamic range and was able to provide higher prediction probabilities as high as 10.0% (2.9-fold higher than chance). ROC curves for the three models showed that they were all reasonable classifiers (Fig 6), with the area under the ROC curve (AUC) being 0.645 for M1, 0.610 for M2, and 0.654 for M3. Thus, while all three of our models were improvements over chance, the model considering eQTL information was most discriminatory and most strongly predicted SNPs that would be prioritized for further functional characterization.

The observation that the complete prediction model (M3) including eQTL information outperformed the two that did not suggested that despite the fact that eQTLs frequently overlap annotated chromatin structures the eQTLs provided additional complementary and independent predictive information regarding the disease relevance of a given SNP. This is confirmed upon inspection of Fig 4, which presents the multivariate adjusted odds ratios (OR) for each feature considered in the M3 model. Strong evidence of association was observed for both eQTL potential (OR = 1.2–2.0 for SNPs with FDR between 0 and < 50%, *P* = 1.2 × 10^−12^ to 7.9 × 10^−91^) and for many of the chromatin states, including those that commonly harbor eQTLs, such as active promoters (#1), a class of strong enhancers (#4), a class of weak enhancers (#7), regions of transcription elongation (#9), and regions of transcription transition (#10) (OR = 1.5–2.3, *P* = 7.1 × 10^−12^ to 1.7 × 10^−145^). Moreover, all of the 78,228 SNP-probe pairs in the two highest bins of probability (with a minimum predicted probability of 6.3% of being a GWAS hit) had both strong evidence for eQTL association and resided in one of these specific functional elements (Table 2). The corresponding 3,291 gene regions originally included 2,067,404 SNP-probe pairs, meaning that for those regions the predictive model reduced by 96.2% the set of eQTLs to inspect further. Of the 78,228 eQTLs, 7,101 (9.1%) were annotated as known GWAS hits, representing 5,644 distinct SNPs and 546 distinct genes. These results suggested that the gain in predictive power in the complete model was a function of the eQTL information to differentiate among SNPs with multiple relevant functional annotations. Furthermore, when we included published scores of predicted SNP pathogenicity from CADD [37] as an additional parameter to M3, there was no substantive change in overall predictive power (M3+CADD, AUC = 0.652) or in the effect estimates of the original predictors (data not shown), reinforcing the notion that our model robustly captured similar information about the disease relevance of non-coding SNPs.

**Table 2:** Functional annotations of SNP-pairs most strongly predicted to be in GWAS loci.

	Meta-analysis FDR bin				
Chromatin state	[0–1%)	[1–5%)	[5–10%)	[10–50%)	[50–100%]
(-) none	0	0	0	0	0
(1) Active Promoter	7,812	244	960	0	0
(2) Weak Promoter	0	0	0	0	0
(3) Poised Promoter	0	0	0	0	0
(4) Strong Enhancer	2,537	0	0	0	0
(5) Strong Enhancer	0	0	0	0	0
(6) Weak Enhancer	0	0	0	0	0
(7) Weak Enhancer	6,703	0	0	0	0
(8) Insulator	0	0	0	0	0
(9) Txn Transition	5,704	1,705	1,317	0	0
(10) Txn Elongation	46,940	144	4,162	0	0
(11) Weak Txn	0	0	0	0	0
(12) Repressed	0	0	0	0	0
(13) Heterochromatin	0	0	0	0	0
(14) Repetitive/CNV	0	0	0	0	0
(15) Repetitive/CNV	0	0	0	0	0

A total of 78,228 SNP-probe pairs (representing 64,495 distinct SNPs and 3,291 distinct genes) were located in the two highest GWAS prediction bins (6.3–10.0%) from the complete prediction model (M3) applied to the testing set. Chromatin states were defined in GM12878 LCLs [12]. CNV, copy number variant; Txn, transcription/transcriptional.

Examining the relative magnitude of the predictive strength of each feature in the complete model provided several important insights regarding the prioritization of candidate SNPs (Fig 4). First, as anticipated, MAF was a significant predictor of GWAS Catalog membership, with the probability of being a GWAS hit being inversely proportional to MAF (OR = 0.17–0.47 for SNPs with MAF ≤ 10%, *P* = 0 to 2.0 × 10^−275^). This was most likely a consequence of the inherent property of GWAS being powered to detect common variants; a biological explanation, however, cannot be dismissed outright.

Second, though chromatin state was an important predictor of GWAS association, we observed substantial heterogeneity within this category of classifiers, including substantial differences between seemingly related states. For example, active promoters were among the strongest predictors (OR = 1.9, *P* = 1.7 × 10^−34^), whereas weak promoters show much weaker association (OR = 1.2, *P* = 5.1 × 10^−02^) and poised promoters provided no discriminatory information (OR = 0.99, *P* = 0.94). Similarly though there were two types of “strong” (#4 and #5) and “weak” (#6 and #7) enhancers, their strengths of association were highly variable (OR = 1.5, *P* = 7.6 × 10^−12^ versus OR = 1.2, *P* = 5.2 × 10^−02^; and OR = 1.3, *P* = 2.1 × 10^−04^ versus OR = 1.6, *P* = 1.0 × 10^−28^, respectively). This observation of the importance of specific chromatin states was further illustrated by the sparseness of Table 2, where notably only two of the four enhancer types (strong #4 and weak #7) were represented in the highest prediction bins. Thus, although similar in annotation, there appeared to be important differences in the disease potentials between related chromatin states.

Third, our model provided greater insight into the relative importance of distance from transcript as a predictor of variant function. Though it is well known that eQTL-associated SNPs are most abundant in close proximity to their target gene [3], the relative impact of distance from transcript as a predictor was relatively modest after multivariate adjustment for eQTL and chromatin state annotations (OR = 1.1–1.2, *P* = 2.5 × 10^−02^ to 5.9 × 10^−11^). To better appreciate the importance of distance as an independent predictor, we ran an additional analysis of the full M3 model that excluded SNP-to-gene distance as a predictor (model “M3B”). In this M3B model, the effect estimates of the other predictors (MAF, eQTL, and chromatin state) were essentially unchanged (data not shown), emphasizing the importance of functional annotations as more direct indicators of relevance. Similarly, in comparing the ROC curves of the two models, we found that the AUC for M3B was 0.652, which was very similar, but slightly lower, than that of the complete model (M3, AUC = 0.654), suggesting that distance provided only marginally more information once other functional annotations were considered. Interestingly, the added value of including distance appeared to be in its influence on discriminatory power at the uppermost part of the predicted probability distribution: the maximum predicted probability observed fell from 10.0% in the full M3 model to 9.1% in the M3B model excluding distance, suggesting that the value of distance as a predictor is in its ability to assist in prioritizing those variants with multiple functional annotations, up-weighting those that are more proximal to genes.

Fourth, we noted the seemingly continuous influence of eQTL potential as a predictor of disease association, even among variants with weak evidence for eQTL association. Though by far the strongest predictors of disease association were for those SNPs with the most significant evidence of eQTL association (i.e., the lower the eQTL FDR, the greater the odds for being a GWAS hit), significant residual association was observed for eQTL variants with FDR = 5–10% (OR = 1.6, *P* = 5.1 × 10^−14^) or 10–50% (OR = 1.2, *P* = 1.3 × 10^−15^), compared to those with FDR > 50% (reference). Nonetheless, the sparseness of Table 2 emphasized that only SNPs with FDR < 10% (and overlapping five specific chromatin states) were found in the highest prediction bins of the full model.

**Fig 7:**
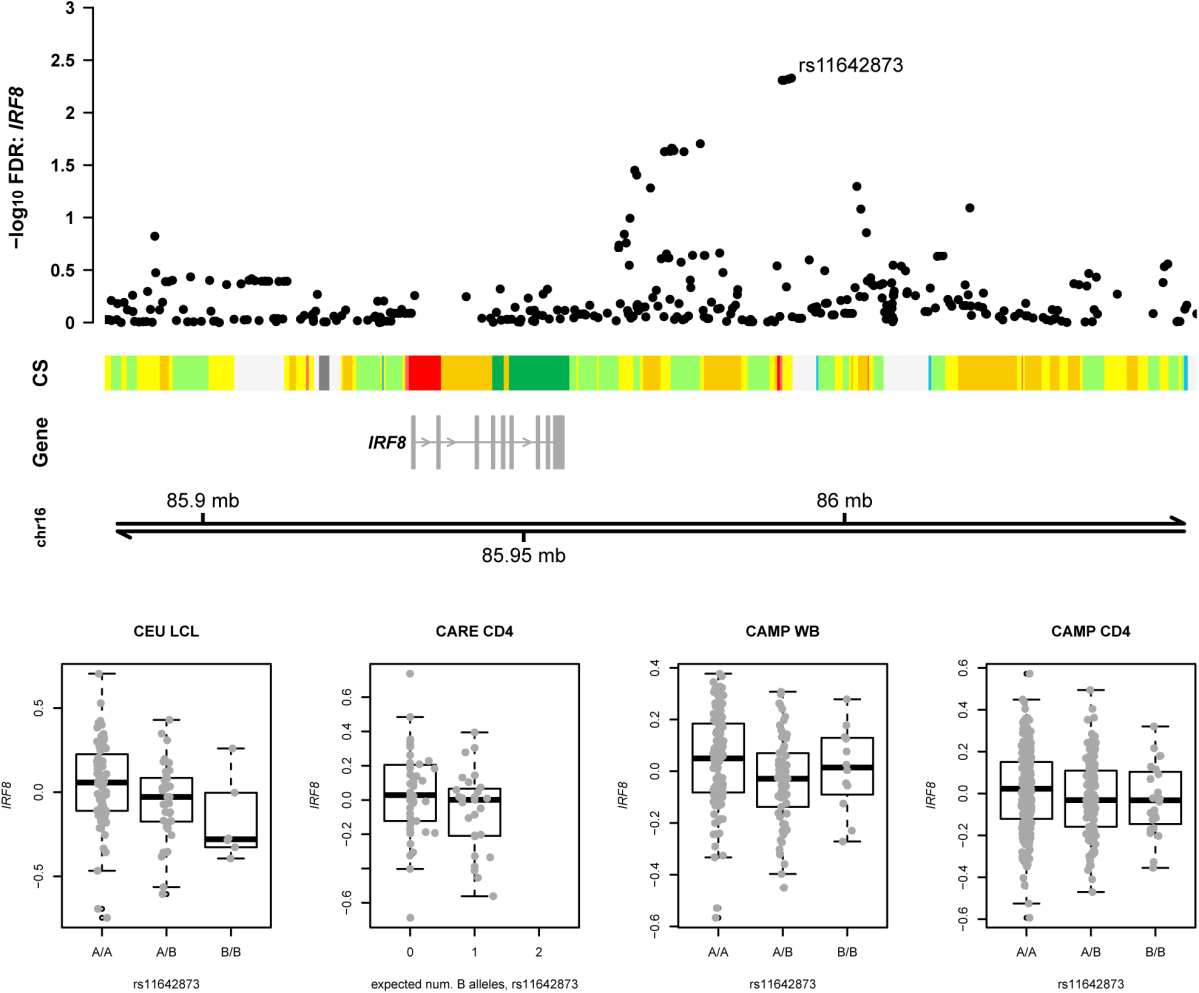
Evidence for an eQTL signal at the *IRF8* locus associated with systemic sclerosis. Top panel:–log_10_ FDR for meta-analysis associations of nearby SNPs with expression of the longer isoform of *IRF8*. Middle panel: Chromatin states (CS) in LCLs (GM12878) [12] and the target *IRF8* transcript. The two most strongly associated SNPs (including the systemic sclerosis GWAS [38] index SNP rs11642873) overlap a predicted weak enhancer region (yellow). Nearby upstream is a predicted active promoter region (red) that is likely spurious given that it overlaps no gene, predicted or otherwise. Bottom panel: boxplots showing probe expression residuals by genotype of index SNP rs11642873 in the four individual cohorts, where the “A” allele is A and the “B” allele is C. None of the cohort-specific associations are individually significant at FDR < 5%, though the meta-analysis is significant at this level.

Finally, we illustrated the components of the predicted probability results in the context of interrogating a particular disease locus to prioritize non-coding variants for further study. Fig 7 shows a genomic region previously identified in a GWAS of system sclerosis (*P* = 2.3 × 10^−12^) mapping near the interferon regulatory factor 8 (*IRF8*) gene [38]. Four polymorphisms in strong LD in this region had previously been reported as an *IRF8* eQTL in a meta-analysis of four HapMap populations (including CEU) [39]. Of these, only one (rs11642873) emerged as a significant candidate in our eQTL meta-analysis. Moreover, this variant resides within a predicted weak/poised enhancer region in LCLs, providing additional functional support. In our predictive model, this SNP had the strongest regional evidence as a disease-associated variant (probability = 6.3%), and indeed was the most strongly associated systemic sclerosis variant reported in the region [38]. This preponderance of data supporting rs11642873 as a functional, disease-associated variant would thus motivate experimental validation of this specific locus.

## Discussion

Distinguishing the relatively small proportion of phenotypically relevant functional non-coding genetic variants from among millions of neutral variants segregating in human populations remains a daunting, yet important, task. Many approaches have been proposed that rely on specific characteristics of the sequence surrounding a variant, including its physical location within known or predicted regulatory regions or transcription factor binding sequence motifs, or the degree of sequence conservation of alleles within or across phyla [6]; yet no single distinguishing feature has emerged that can be confidently relied upon. The recent availability of rich genomic sequence annotations of chromatin marks via projects such as ENCODE [4], and the recognition of specific chromatin states that correspond to local transcriptional activity represent an important further advance [12], together with multiple publications demonstrating significant enrichment for regulatory polymorphisms among GWAS-identified variants [2, 3, 9], provide incentive to consider the joint effects of these different sources of information.

Here, we demonstrate the value of combining multiple lines of information to improve our predictive capabilities for identifying disease-associated genetic candidates. We leveraged the power of eQTL meta-analysis in combination with other available genomic SNP annotations to develop logistic multivariate models to predict disease relevance. Our complete M3 model that incorporated information on eQTL associations along with chromatin states outperformed the others, enabling better discrimination and wider separation of probability estimates between variants, particularly through the identification of a subset of variants most strongly enriched for GWAS hits. Most notable, even when combined with other SNP annotations that are correlated with it, eQTL association information enhanced the ability of our predictive model to prioritize variants. The observation that even moderate to modest evidence for an eQTL (FDR = 5–50%) was strongly and significantly predictive suggested that although hard significance threshold cutoffs do provide for greater confidence for those variants that are given highest prioritization, the arbitrary setting of such cut-points may result in the loss of valuable information when attempting to prioritize non-coding genetic variation. Considering the known enrichments for eQTLs and for GWAS SNPs in chromatin-regulated regions (summarized in [40]), it would not have been surprising if chromatin state information alone would have been sufficiently predictive. While the dynamic range of the M2 model was slightly reduced compared to M1 upon including chromatin state, the biological interpretability of its predictions were qualitatively improved, being able to suggest mechanistic hypotheses beyond what a simple distance measure can provide. The evolution of the prediction model also identified two specific classes of enhancer chromatin states as meriting closer attention in forming those hypotheses. Overall, the extent to which the inclusion of eQTL information improved model prediction, particularly for the subset of variants with the highest probabilities of being disease-associated, was thus particularly striking, and further implicates regulatory potential as an important determinant of the phenotypic potential of a variant.

Our work further highlights the ongoing need to more fully catalog the spectrum of regulatory genetic variation in human populations, and the potential benefit of applying meta-analytic approaches in this regard. The gains in eQTL detection achieved by our initial meta-analysis clearly illustrate this benefit. In our study, of the 788 genes identified only by eQTL meta-analysis, only 28 (3.6%) harbor at least one previously disease- or trait-associated SNP. However, among these 788 eQTL genes, our predictive model identified no fewer than 567 SNPs with the highest predicted probability of being GWAS hits (predicted probabilities > 6.3%), corresponding to 138 genes (24%). Examining the entire testing set, of the 78,228 SNP-probe pairs with such high prediction probabilities, only a fraction (7,101 or 9.1%) are currently present in the NHGRI GWAS Catalog; the remainder may therefore represent plausible candidates simply not yet implicated by GWAS. These should be considered as priorities for consideration in reinterpretation of available GWAS datasets. To help facilitate such efforts, we have made the full set of prediction scores, together with the eQTL meta-analysis results, available for download.

The predictive scores can also be used to prioritize variants for functional characterization, including the prioritization of tightly linked variants within a disease-associated region that all demonstrate similar evidence of disease association. In such scenarios, the ability to rank order variants based on their functional characteristics and their likelihood of being disease-associated may help reduce the number of loci to be evaluated functionally to a manageable handful. We have highlighted one such example at the *IRF8* GWAS locus for systemic sclerosis, but a more systematic examination of the data motivated by interests in particular diseases and/or traits is needed. Thus, an important future application of this predictive model work is to apply such prioritization metrics to published GWAS results, analogous to a method for using eQTL evidence as weighted priors to improve statistical power when re-analyzing GWAS data [41].

While the work presented here illustrates a potentially useful approach for the enhanced identification of disease-associated variants, the fact that our highest probability estimates peak at 10.0% highlights the need for further improvements. To this end, we recognize some limitations of the current model that if addressed may improve its predictive capabilities. Foremost would be the extension to eQTLs and chromatin state data representative of the diverse set of tissues and cell types. Our current model includes eQTL data from whole blood, circulating CD4+ lymphocytes, and LCLs–a rather narrow spectrum of related cell types. While potentially sufficient for ranking variants related to immune or inflammatory disorders, consideration of eQTL data from other tissues and cells (including, liver, adipose, and neurological tissue) may help improve the ability of the model to predict susceptibility variants for diseases of those organs [18]. The strong enrichment of our eQTL results for immune and metabolic traits, but not for height- or cancer-related variants supports this possibility. Extensions of the meta-analytic eQTL approach to explicitly model cell-type specificity have been proposed to gain additional statistical power to detect eQTLs [16–18]. Our approach to eQTL enumeration may also have some biases owing to the use of association yield optimization to choose latent factors for adjustment in the association model. Finally, in review, we were made aware of a recent publication describing an analogous and comparable logistic regression model for predicting SNP disease relevance [42], which illustrates how our differing choices in model construction related to source annotations (e.g., blood eQTLs vs. blood+brain+liver eQTLs; summary chromatin states vs. individual genomic marks) and statistical methodologies (e.g., standard vs. regularized logistic regression; size and membership of testing and training sets) indeed tune the prediction problem for all disease-associated variants and for specific sub-groups of them.

Another inherent limitation of our methodological approach to the question of predicting the disease relevance of genetic variants was our reliance on the GWAS Catalog for the training and testing of our models. Though fairly comprehensive in its inclusion of a very large proportion of published associations detected by GWAS, the Catalog only summarizes associations for those variants specifically discussed in published manuscripts. More accurate representations of the disease association propensity of a variant would come from consideration of deeper sets of phenotypic association results, rather than limiting inclusion to the most strongly associated variants and reliance on the reporting practices of individual research groups. To do so would require access to the summary statistics for all variants across all studies. Though summary statistics are available for a subset of published GWAS, the GWAS Catalog includes a much larger sampling of disease phenotypes. Thus, our focus on the GWAS Catalog as a reference represents a tradeoff between breadth (the diverse number of diseases and traits represented in the GWAS Catalog) and depth (the complete set of summary statistics for a smaller subset of GWAS). Furthermore, the GWAS Catalog is biased against lower-frequency SNPs because GWAS are inherently underpowered to detect significant associations for low-frequency variants. This skewing towards more common variants could certainly influence the predictive characteristics of our model. Despite these limitations, our results should motivate additional efforts to characterize the regulatory and disease potential of genetic variation.

In summary, we have demonstrated the utility of combining eQTL association data across multiple populations and tissue types to increase power for eQTL detection and the additional value in leveraging that information to prioritize disease-associated non-coding SNPs for further functional characterization.

## Supporting Information

S1 Fig Quantile-quantile plot of meta-analysis eQTL associations shows substantial enrichment of associations. For each of 7,672,940 SNP-probe pairs, position on the *y*-axis is the observed *χ* ^2^ test statistic and position on the *x*-axis is the expected *χ* ^2^ test statistic based on a permuted distribution. For clarity, the count of SNP-probe pairs within a given range bin of observed *χ* ^2^ test statistics are shown rather than plotting each individual point. For example, the first count in the bottom left-hand corner is the count of pairs in the range 0 ≤ *χ* ^2^ < 1.3 and the last count in the top right-hand corner is in the count in the range *χ* ^2^ ≥ 87.9. The horizontal lines represent different thresholds of false discovery rates (0.1%, 0.5%, 1%, and 5%).
(TIF)

S2 Fig Forest plot of component effects of two smaller GWAS predictive models based on training set of SNPs. Odds ratios (black squares) for model features predicting the membership of a SNP in the NHGRI GWAS Catalog are shown here with standard errors (gray lines). The complete multivariate model is shown in Fig 4. There are three classes of SNP annotation represented in each of the two models, each with multiple levels: distance from the transcript boundaries of its target gene, its MAF, and its gene structural classification (“structure [M1]”, top panel) or predicted chromatin state in GM12878 LCLs [12] (“chromstate [M2]”, bottom panel). The base levels for each annotation are “0 kb (within gene)” [Distance from Gene], “>10%” [MAF], and “none” [Structural Annotation] or “Heterochromatin (13)” [ChromHMM].
(TIF)
