# Supplementary figures and images for: Expression Quantitative Trait Loci Information Improves Predictive Modeling of Disease Relevance of Non-Coding Genetic Variation
Source: PLoS One. 2015 Oct 16;10(10):e0140758. doi: 10.1371/journal.pone.0140758 (PMC4608673; doi:10.1371/journal.pone.0140758)

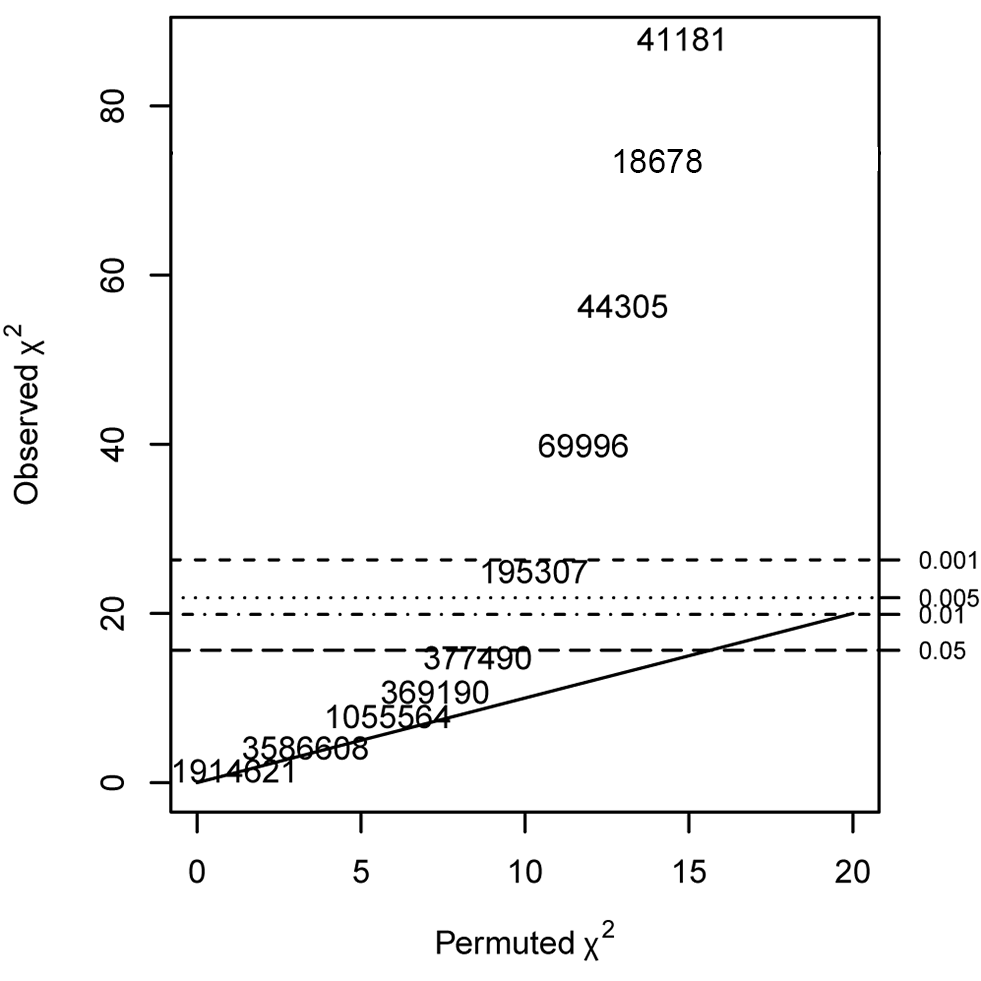

Supplement: S1 Fig — For each of 7,672,940 SNP-probe pairs, position on the y-axis is the observed χ 2 test statistic and position on the x-axis is the expected χ 2 test statistic based on a permuted distribution. For clarity, the count of SNP-probe pairs within a given range bin of observed χ 2 test statistics are shown rather than plotting each individual point. For example, the first count in the bottom left-hand corner is the count of pairs in the range 0 ≤ χ 2 < 1.3 and the last count in the top right-hand corner is in the count in the range χ 2 ≥ 87.9. The horizontal lines represent different thresholds of false discovery rates (0.1%, 0.5%, 1%, and 5%). (TIF) [file pone.0140758.s001.tif]

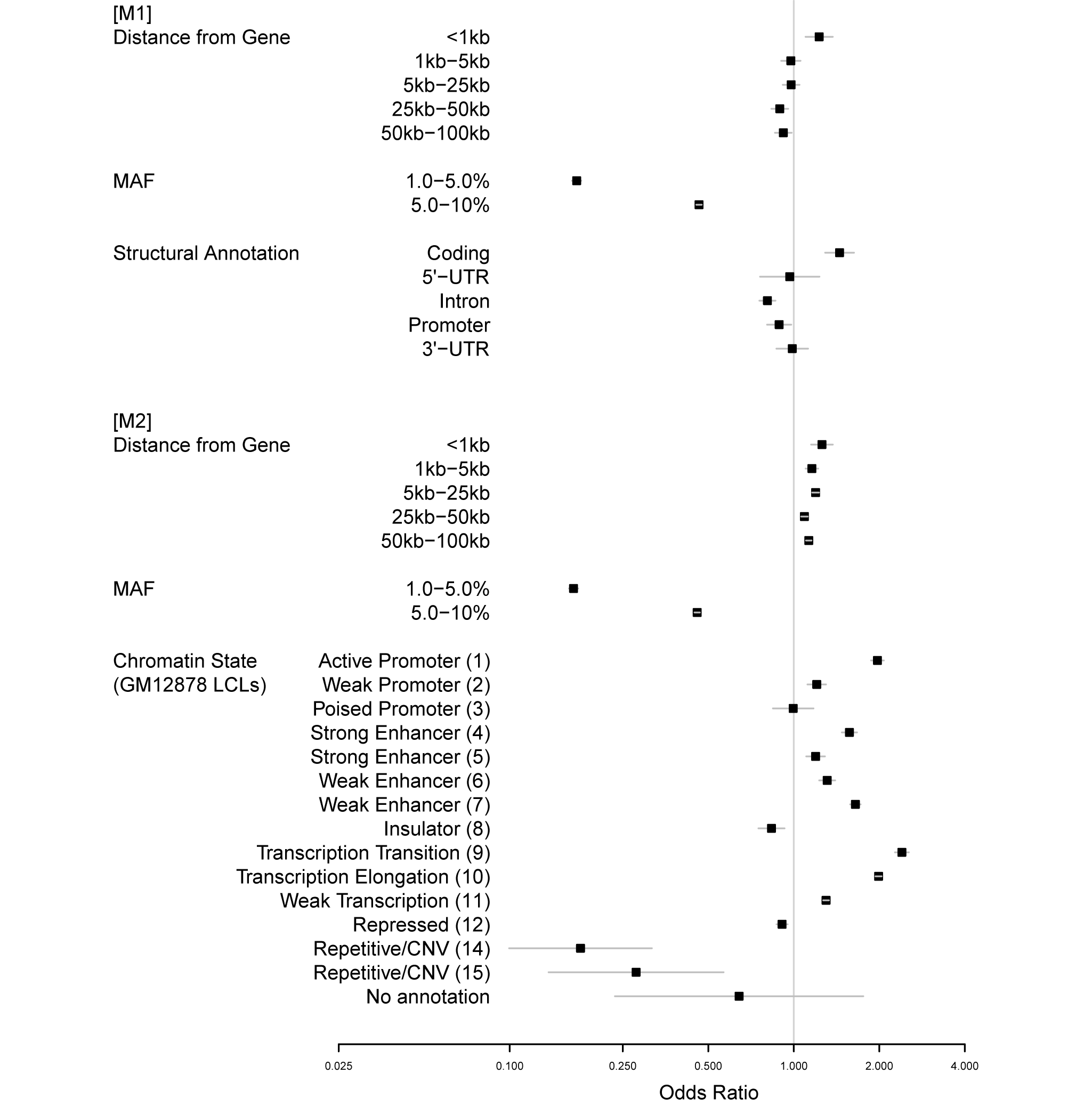

Supplement: S2 Fig — Odds ratios (black squares) for model features predicting the membership of a SNP in the NHGRI GWAS Catalog are shown here with standard errors (gray lines). The complete multivariate model is shown in Fig 4. There are three classes of SNP annotation represented in each of the two models, each with multiple levels: distance from the transcript boundaries of its target gene, its MAF, and its gene structural classification (“structure [M1]”, top panel) or predicted chromatin state in GM12878 LCLs [12] (“chromstate [M2]”, bottom panel). The base levels for each annotation are “0 kb (within gene)” [Distance from Gene], “>10%” [MAF], and “none” [Structural Annotation] or “Heterochromatin (13)” [ChromHMM]. (TIF) [file pone.0140758.s002.tif]
